# Zinc level and its impact on the phenotype of sickle cell disease

**DOI:** 10.1007/s00277-025-06429-4

**Published:** 2025-06-04

**Authors:** Ilham Youssry, Mona El-Ghamrawy, Eman R. Youness, Nahla A. Mohamed, Yasmeen Mohammed Mahmoud Selim

**Affiliations:** 1 Department of Pediatrics, Pediatric Hematology and Bone Marrow Transplantation, Kasr Alainy Faculty of Medicine, Cairo University, 2 Tayseer Street, End of King Faisal Street, Giza, Egypt; 2 Department of Medical Biochemistry, Medical Research Division, National Research Centre, Giza, Egypt; 3 Department of Pediatrics, El-Galaa Teaching Hospital, Cairo, Egypt

**Keywords:** Sickle cell disease, Zinc deficiency, Clinical phenotype, Oxidative status

## Abstract

Zinc is an important antioxidant, and its deficiency contributes to oxidative damage in sickle cell disease (SCD). Emerging evidence supports zinc’s beneficial effects on SCD phenotype. This study aimed to determine the prevalence of zinc deficiency and assess the impact of zinc supplementation on clinical, hematological, and oxidative parameters in SCD children. Sixty SCD children were enrolled in this single-arm prospective study. All participants received daily oral zinc sulfate for 12 months [10 mg for ages 4–8, 20 mg for ages 9–13, and 30 mg for ages 14–18]. Clinical assessments and laboratory evaluations, including serum zinc, copper, nitric oxide, and total antioxidant activity, were conducted at baseline and after 12 months. The patients’ mean age was 9.2 ± 4.1 years; 80% had HbSS and 20% had sickle β-thalassemia. All patients were zinc-deficient at baseline (mean zinc: 42.8 ± 14.1 µg/dl). Post-supplementation, there were significant improvements in weight and height Z-scores (p-values < 0.001), and reductions in the frequency of vaso-occlusive crises, infections, hospitalizations, and transfusions (p-values < 0.001). Significant increases were observed in hemoglobin, hematocrit, fetal hemoglobin, zinc, nitric oxide, and total antioxidant activity (p-values < 0.001, < 0.001, 0.001, < 0.001, < 0.001, and < 0.001 respectively), while serum bilirubin, ferritin, and copper levels decreased significantly (p-values = 0.029, < 0.001, and 0.009, respectively). Zinc deficiency appears highly prevalent among SCD children, and supplementation may offer clinical, hematological, and antioxidant benefits. This study highlights the potential beneficial effects of zinc in this high-risk population.

## Introduction

Zinc is a micronutrient of paramount importance in the human body, with substantial involvement in physiological and pathological processes. Zinc is necessary for cellular growth, tissue differentiation, and is incorporated in over 300 enzymes regulating physiological processes, such as anti-inflammatory, antioxidant, and immunological responses [1, 2]. Zinc is typically known for its antioxidant properties at adequate levels. However, like other antioxidants, zinc can exhibit pro-oxidant effects in severe deficiencies or when present in excess [3]. Zinc deficiency promotes oxidative stress as zinc is a cofactor for the antioxidant defense system, optimizing the function of superoxide dismutase, a scavenging enzyme essential to detoxify superoxide anion to hydrogen peroxide and thus controlling free radicals’ generation [4]. Zinc also prevents cellular oxidative damage through membrane stabilization, inhibition of nicotinamide adenine dinucleotide phosphate oxidase (NADPH-Oxidase), which is a pro-oxidant enzyme, and induces the formation of metallothionein, which lowers hydroxyl radicals (OH) and sequesters reactive oxygen species (ROS) [5, 6]. Zinc excess promotes oxidative stress by disrupting the redox balance, impairing mitochondrial function, and dysregulating intracellular signaling pathways involved in apoptosis and inflammation [3, 7]. Copper is another cofactor in the antioxidant defense system required for the superoxide dismutase activity, however, copper acts as a pro-oxidant in high concentrations. Zinc bioavailability influences copper levels in the body; a zinc shortage enhances copper absorption in the gut, with increased copper levels, potentiating oxidative stress [8–12], and a zinc excess displaces other trace metals (e.g., copper, and iron) from metalloproteins, which can then participate in Fenton-like reactions, generating reactive oxygen species (ROS) [13].

Several earlier studies have documented zinc deficiency among patients suffering from sickle cell disease (SCD), with the etiology of this deficiency being multifactorial, including inadequate dietary intake, poor absorption, increased requirements due to chronic hemolysis, increased urinary losses from renal insufficiency, or increased intestinal losses due to increased intestinal permeability from VOC-associated intestinal injury [14–20]. Chronic inflammation may also contribute to zinc deficiency in SCD. Pro-inflammatory cytokines, such as IL-6, induce hepatic metallothionein synthesis, which can sequester zinc intracellularly and reduce circulating levels. This redistribution is part of the acute phase response and may play a role in the hypozincemia observed in chronic inflammatory states such as SCD [21]. This deficiency aggravates many pathophysiologic changes in SCD. It is related to many complications related to the disturbance in the antioxidant defense system, a condition previously described in SCD [22–24]. Furthermore, there is growing evidence of the beneficial impact of zinc supplementation on the clinical phenotype of SCD [25–28].

The objective of this study was to determine the prevalence of zinc deficiency and its correlation with the clinical phenotype of children with SCD and to determine the impact of oral zinc supplementation on the clinical, hematological, and oxidative status of zinc-deficient SCD children.

## Methods

### Study design and study population

This prospective study included 60 SCD children who met the inclusion and exclusion criteria, recruited from Cairo University’s Pediatric Hematology Outpatient Clinic. The inclusion criteria were any steady-state SCD patient (including homozygous sickle cell anemia [HbSS] and sickle β-thalassemia [HbSβ^0^/HbSβ^+^]) aged ≤ 18 years. Steady state was defined as the absence of any acute episodes (e.g., infection, vaso-occlusive crisis, stroke, and acute chest syndrome) for at least four weeks before enrollment. Patients excluded from the study were those who received blood transfusion ≤ 4 weeks before enrollment, those with associated conditions known to affect zinc levels such as acrodermatitis enteropathica, and diarrheal diseases, and those taking dietary supplements containing zinc at any time before enrollment.

The Research Ethics Committee at Cairo University’s Faculty of Medicine approved this study. The written informed consent and assent were obtained from the patients and their legal guardians. All procedures were carried out following the 1964 Helsinki Declaration, as well as any subsequent amendments or similar ethical norms.

### Treatment schedule

All enrolled zinc-deficient patients were assigned to receive zinc sulfate oral therapy (zinc sulfate 20 mg/5 ml syrup, Sulfozinc, Chemical Industries Development Company [CID Co.], Egypt), as a single daily dose for 12 months. The zinc sulfate doses dispensed to patients according to their age were as follows: 10 mg of elemental zinc for those aged 4 to 8 years, 20 mg of elemental zinc for those aged 9 to 13 years, and 30 mg of elemental zinc for those aged 14 to 18 years. These doses have been based on the existing knowledge of the Recommended Dietary Allowances (RDAs) and Tolerable Upper Intake Levels (ULs) for zinc, and its safety profile in children according to age [29]. The Sulfozinc syrup was orange-flavored. The syrup was taken on an empty stomach (1 h before breakfast for optimal absorption). A 3-month supply of the syrup was provided to patients, and instructions were given to keep the bottles in a dry place, at room temperature, not exceeding 30ºC, as per the manufacturer’s instructions.

### Schedule of assessment

At baseline, all enrolled patients underwent a detailed physical examination encompassing anthropometric measurements (weight and height), and relevant history of SCD-related complications, including the frequency and severity of vaso-occlusive crises (VOCs), the frequency of infections, the frequency of blood transfusions, and the frequency of hospitalizations during the preceding 12 months. All concomitant medications were obtained. Titration of the hydroxycarbamide to the maximum tolerated dose was not allowed during the study. A VOC event was defined as any pain in the head, chest, abdomen, back, or extremities with no other identifiable cause [30]. Severe vaso-occlusive crises were defined as events requiring a visit to the emergency department or physician’s office, or extremely painful events requiring hospitalization [31]. All baseline data were retrieved from the patients’ archived files or by direct patient interviewing.

All patients underwent follow-up visits at 3, 6, 9, and 12 months after the initiation of zinc replacement to replenish the zinc supply, assess adherence (compliance) to therapy, evaluate adverse events, and undergo a detailed physical examination.

Adherence (compliance) was defined as the degree to which a patient follows the dosage schedule and the recommended interval. Compliance was assessed over a period of time and expressed as a percentage [32]. Several methods were used to evaluate adherence to the study protocol. Monthly calendars and stickers were given to all patients and caregivers. To indicate which days they took the supplement, they were instructed to put the stickers on the calendar. Caregivers were instructed to bring back the syrup bottles every 3 months to be exchanged for new bottles for the following 3 months; residual volumes in the returned bottles were measured. Finally, interviews were conducted during each visit to gather information regarding the frequency of supplement use. The research team analyzed this information and ranked compliance levels as follows: Supplement usage: ≥75%, < 75%, or unclear.

### Laboratory evaluations

Complete blood count (CBC) with blood indices, reticulocyte count, renal function tests (blood urea and serum creatinine), liver function tests (alanine aminotransferase [ALT], aspartate aminotransferase [AST], total serum bilirubin [TSB], and direct serum bilirubin [DSB]), serum ferritin, lactate dehydrogenase (LDH), fetal and sickle hemoglobin by hemoglobin electrophoresis, serum zinc, serum copper, and antioxidant markers (serum nitric oxide [NO] and total antioxidant activity [TAOA]) were evaluated for all patients at baseline and at 12 months. Safety laboratory assessments, including CBC, renal, and liver function tests were done every 3 months as per the standard of care for SCD patients.

### Sample collection and materials

Each patient had five milliliters of venous blood drawn and placed into ethylenediaminetetraacetic acid (EDTA) tubes. Morning non-fasting samples were obtained. Samples were withdrawn at baseline and 12 months (Baseline and end-of-study samples were withdrawn at least 4 weeks from blood transfusion). The samples were centrifuged at 3000 rpm for 10 min after a clot formed at room temperature. After that, the serum was collected and either examined or kept for later analysis in a freezer set at -80° C.

### Serum zinc and copper determination

*A BUCK SCINTIFIC 210/211 VGP VER3.94 C* atomic absorption spectrophotometer was used to measure the zinc and copper levels in the serum. Serum zinc and copper levels were expressed in µg/dl.

We adopted the International Zinc Nutrition Consultative Group (IZiNCG) definition of low serum zinc levels at a cut-off value of 65 µg/dl for children < 10 years old (whatever their gender), 66 µg/dl for girls ≥ 10 years old and 70 µg/dl for boys ≥ 10 years old [33]. Serum copper reference ranges were 75–153 µg/dl for children under 10.3 years, 64–132 µg/dl for those between 10.3 and 12.5 years, and 57–129 µg/dl for those beyond 12.5 years [34].

### Nitrite determination

Nitrite was measured using an assay based on a diazotization reaction initially described by the Griess test [35]. The chemical reaction that employs sulfanilamide and N-L-naphthyl ethylenediamine dihydrochloride (NED) in an acidic (phosphoric acid) environment is the basis for the Griess Reagent System. This system is capable of detecting nitrite in a range of biological and experimental liquid matrices, including plasma. The average absorbance of the triplicates of each experimental sample was recorded. Each experimental sample’s nitrite concentration (Y) was estimated by comparing it to the Nitrite Standard reference curve. The standard curve-generated formula, *Y = 0.0185X + 0.106*, was applied, where X represents the experimental sample’s average absorbance. Levels of nitric oxide were reported in µM/ml.

### Total antioxidant activity (TAOA) determination

The method developed by *Koracevic and his colleagues* was used to determine the total antioxidant activity (TAOA). The assay quantifies the degree to which the free oxygen radicals generated by Fenton’s reaction hinder the synthesis of Thiobarbituric Acid Reactive Substances (TBARS) from sodium benzoate. The standard was a 1 mmol/L uric acid solution. Hydroxyl radicals are produced when a standardized solution of Fe-EDTA complex combines with hydrogen peroxide in a Fenton-type reaction. The degradation of benzoate by the reactive oxygen species (ROS) leads to the release of TBARS. The added sample’s antioxidants suppress the production of TBARS. Malondialdehyde (MDA) easily engages with 2-thiobarbituric acid (TBA) in a nucleophilic addition reaction at low pH and high temperature (90–100 °C), producing a red, luminous 1:2 MDA: TBA adduct. The reaction is measured either colorimetrically at 530–540 nm or fluorometrically at 530 and 550 nm excitation and emission wavelengths, respectively [36–38].

### Statistical analysis

IBM SPSS (Statistical Package for the Social Sciences; IBM Corp., Armonk, NY, USA), version 24 for Microsoft Windows, was used for all statistical calculations. Continuous variables were tested for normality using the Shapiro-Wilk test and presented as mean ± standard deviation, median, and range. For comparisons between pre- and post-intervention values, we used the paired t-test for normally distributed continuous variables, and the Wilcoxon signed-rank test for non-normally distributed or ordinal data. Categorical variables were analyzed using the McNemar test or the Chi-square test, as appropriate. All frequency data, including values of zero, were retained and treated as valid outcomes. The non-parametric tests applied were robust to zero values and did not require data transformation or exclusion. Negative Binomial regression analysis was used to model relationships involving clinical count data, as it was suitable for non-normally distributed count outcomes. This approach allowed us to estimate incidence rate ratios (IRRs) with corresponding 95% confidence intervals (CIs) and p-values, providing interpretable measures of association between continuous predictors and event frequencies. A p-value of ≤ 0.05 was deemed statistically significant [39–41].

## Results

Forty-eight (80%) of our studied SCD children were homozygous (HbSS), and 12 (20%) were compound heterozygous for sickle β-thalassemia (HbSβ). Thirty-four (56.7%) were males, and 26 (43.3%) were females. The mean age at diagnosis was 3.2 ± 2.1 years, and the mean age at enrollment was 9.2 ± 4.1 years. All our studied patients were receiving vitamin D3 supplementation: cholecalciferol (1000–3000 IU/day), calcium carbonate (1000–2000 mg/day), folic acid (5 mg/day), L-carnitine (50 mg/kg/day), and penicillin prophylaxis. The majority of our patients (54; 90%) received hydroxycarbamide at a mean daily dose of 22.7 ± 5.2 mg/kg/day, with dose adjustments for changes in weight or toxicities. Thirteen (21.7%) patients received iron chelation for iron overload; the mean serum ferritin level was 1265 ± 1016 ng/ml. At baseline, the mean hematological parameters of the studied patients were as follows: hemoglobin was 8.6 ± 1.5 gm/dL, platelet count was 358 ± 209 × 10^3^/cmm, and total leukocyte count was 10.4 ± 5.1 × 10^3^/cmm. The mean sickle hemoglobin (HbS) level was 70.0 ± 12.8%, and the mean fetal hemoglobin (HbF) level was 18.2 ± 9.2%.

All our studied patients had low serum zinc levels with a mean of 42.8 ± 14.1 µg/dl (Range: 13–62 µg/dl). All our studied patients had normal serum copper levels according to age, with a mean of 97.5 ± 38 µg/dl (Range: 57–199 µg/dl). The mean NO level was 6.8 ± 3.3 µM/ml (Range: 1.4–19.4 µM/ml), and the mean total antioxidant activity was 0.8 ± 0.3 mmol/l (Range: 0.1–1.6 mmol/l) as illustrated in Table 1.

**Table 1:** Zinc, copper, nitric oxide, and total antioxidant activity in the studied SCD patients (*n* = 60)

Measured Variables		Value
Zinc (µg/dl)	Mean ± SD.	42.8 ± 14.1
	Median (Range)	46 (13–62)
Copper (µg/dl)	Mean ± SD.	97.5 ± 38
	Median (Range)	91 (57–199)
NO (µM/ml)	Mean ± SD.	6.8 ± 3.3
	Median (Range)	6 (1.4–19.4)
Total antioxidant activity (mmol/l)	Mean ± SD.	0.8 ± 0.3
	Median (Range)	0.8 (0.1–1.6)

SD: Standard deviation, NO: Nitric oxide

*Association between baseline serum zinc levels and clinical outcomes*

**Fig. 1:**
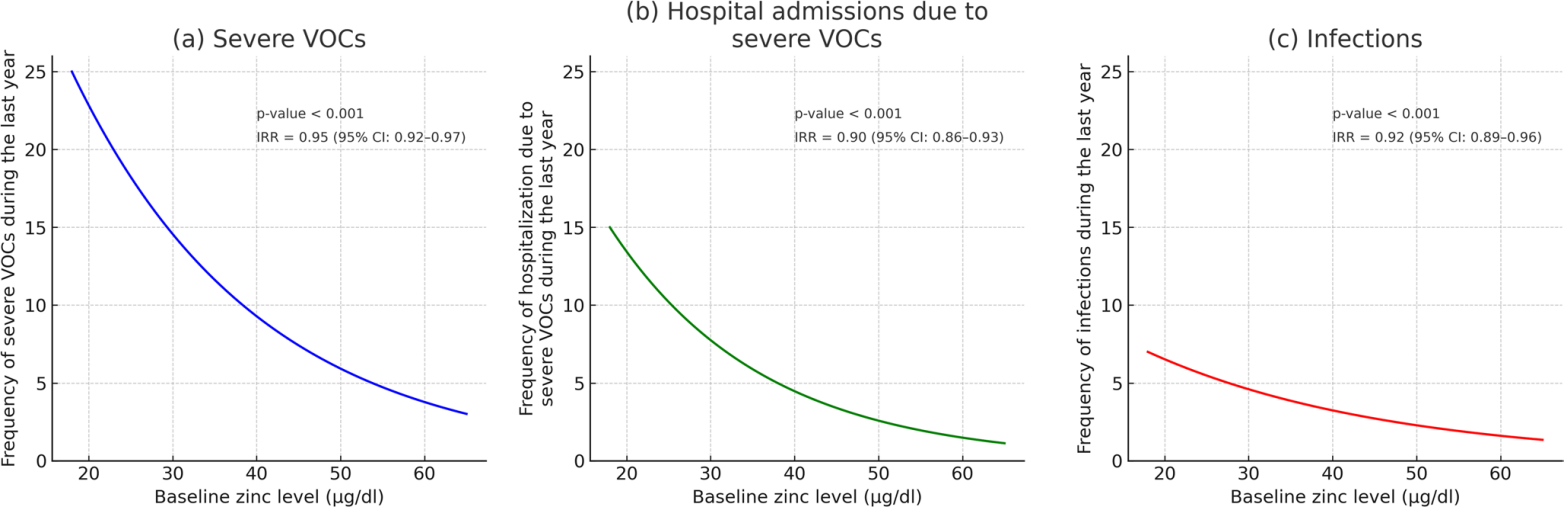
Predicted associations between baseline zinc level and clinical outcomes using Negative Binomial Regression. Each panel displays the model-predicted frequency of (a) severe vaso-occlusive crises (VOCs), (b) hospital admissions due to severe VOCs, and (c) infections. Incidence rate ratios (IRRs), 95% CI (confidence intervals), and p-values are annotated.

Negative Binomial Regression analyses revealed significant inverse associations between baseline serum zinc level and the frequency of severe VOCs, hospital admissions due to severe VOCs, and infections during the preceding 12 months as follows; higher zinc levels were inversely associated the frequency of severe VOCs (IRR = 0.95, 95% CI: 0.92–0.97, p-value < 0.001), the frequency of hospital admissions due to severe VOCs (IRR = 0.89, 95% CI: 0.86–0.93, p-value < 0.001) and the frequency of infections (IRR = 0.92, 95% CI: 0.89–0.96, p-value < 0.001). These associations are illustrated in Fig. 1 (a, b, and c).

On average, 95% of patients were compliant with zinc supplementation (supplement taken ≥ 75% of the time). No serious zinc-related adverse events have been reported. The only reported zinc-related adverse events were constipation in 6 patients (10%) and nausea in 3 patients (5%), and neither necessitated any dose interruption or adjustment.

Regarding anthropometric measurements of the studied patients, there was a significant improvement in weight and height Z scores by the end of the study (p-values < 0.001). Also, there was a significant decrease in the markers of disease severity, including the total number of VOCs, the number of severe crises, infection rate, hospitalization rate, as well as the length of hospital stays, after zinc supplementation (p-value < 0.001, < 0.001, < 0.001, < 0.001, and 0.001 respectively). Furthermore, the frequency of blood transfusions decreased after zinc supplementation (p-value < 0.001), as illustrated in Table 2.

Hematological improvement after zinc replacement was approved by the significant increase of the hemoglobin and hematocrit by the end of the study (p-value < 0.001, and < 0.001, respectively). Fetal hemoglobin, zinc level, nitric oxide level, and total antioxidant activity also showed a significant increase after zinc replacement (p-value = 0.001, < 0.001, < 0.001, < 0.001, respectively). Moreover, the total serum bilirubin, serum ferritin, and serum copper levels showed a significant decline by the end of the study (p-value = 0.029, < 0.001, and 0.009, respectively) as illustrated in Table 2.

**Table 2:** Comparison of the clinical, hematological, and oxidative status of the studied SCD patients (*n* = 60) before and 12 months after zinc supplementation

Studied Variables	Baseline (Before Zinc Supplementation)	End of Study (After 12-month Zinc Supplementation)	*p*-value
Anthropometric measurements (Mean ± SD.)			
Weight Z score	− 0.66 ± 0.75	− 0.69 ± 0.48	*< 0.001*
Height Z score	− 1.44 ± 1.24	− 1.34 ± 0.99	*< 0.001*
Frequency of VOCs during the preceding 12 months [Median (range)]	12 (0–28)	6 (0–16)	*< 0.001*
0 (*n*, %)	7 (11.7%)	14 (23.3%)	*< 0.001*
1–10 (*n*, %)	21 (35.0%)	34 (56.7%)	
11–20 (*n*, %)	22 (36.7%)	12 (20.0%)	
> 20 (*n*, %)	10 (28.6%)	0 (0.0%)	
Frequency of Severe VOCs during the preceding 12 months [Median (range)]	2 (0–12)	0 (0–7)	*< 0.001*
0 (*n*, %)	19 (31.7%)	41 (68.3%)	*< 0.001*
1–3 (*n*, %)	24 (40.0%)	19 (31.7%)	
> 3 (*n*, %)	17 (28.3%)	0 (0.0%)	
Number of patients experiencing infections (n, %)	35 (58.3%)	21 (35.0%)	*0.004*
Frequency of infections during the preceding 12 months [Median (range)]	1 (0–6)	0 (0–3)	*< 0.001*
Frequency of blood transfusion during the preceding 12 months [Median (range)]	3.5 (1.0–12.0)	2.0 (1.0–6.0)	*< 0.001*
Frequency of hospital admissions during the preceding 12 months [Median (range)]	3 (0–12)	0 (0–7)	*< 0.001*
Length of hospital admission (days) [Median (range)]	16 (1–58)	9 (1–26)	*0.001*
Laboratory parameters (Mean ± SD.)			
Hemoglobin (gm/dl)	8.6 ± 1.5	9.3 ± 1.4	*< 0.001*
Hematocrit (%)	24.5 ± 4.8	26.4 ± 3.1	*< 0.001*
Red blood cell count (x10^6^/cmm)	3.0 ± 0.8	3.1 ± 0.6	0.089
MCV (fl.)	83.3 ± 13.5	83.5 ± 12.4	0.573
PLT (×10^3^/cmm)	358 ± 209	324 ± 176	0.197
TLC (×10^3^/cmm)	10.4 ± 5.1	10.5 ± 5.2	0.982
Reticulocyte count (%)	7 ± 5.3	6.5 ± 5.7	0.717
TSB (mg/dl)	2.1 ± 1.8	1.6 ± 0.7	*0.029*
DSB (mg/dl)	0.32 ± 0.17	0.3 ± 0.2	0.092
AST (u/l)	38.78 ± 26.77	37.95 ± 18.07	0.375
LDH (u/l)	371.14 ± 164.65	412.06 ± 150.13	0.166
Serum Ferritin (ng/ml)	1265 ± 1016	959.01 ± 134.41	*< 0.001*
HbS (%)	70.0 ± 12.8	70.2 ± 10.9	0.721
HbF (%)	18.2 ± 9.2	26.92 ± 17.65	*0.001*
Serum Zinc (µg/dl)	42.8 ± 14.1	94.1 ± 18.4	*< 0.001*
Serum Copper (µg/dl)	97.5 ± 38	88.3 ± 41.3	*0.009*
Serum NO (µM/ml)	6.8 ± 3.3	9.1 ± 3.3	*< 0.001*
Serum Total antioxidant activity (mmol/l)	0.8 ± 0.3	1.2 ± 0.2	*< 0.001*

VOC: Vaso-occlusive crisis, MCV: Mean corpuscular volume, PLT: Platelet count, TLC: Total leucocyte count, TSB: Total serum bilirubin, DSB: Direct serum bilirubin, AST: Aspartate aminotransferase, LDH: Lactate dehydrogenase, HbS: Sickle hemoglobin, HbF: Fetal hemoglobin, NO: Nitric oxide

## Discussion

In this study, low serum zinc levels have been demonstrated in all the studied SCD children, yielding a prevalence of 100%. Several researchers previously demonstrated low levels of zinc in SCD children with varying prevalences, as low as 44% in the study conducted by *Leonard et al.* [42], as high as 96% in the study conducted by *Edbor et al.* [43], and approximately 60–65% in the ZIPS (Zinc for Infection Prevention in Sickle Cell Anemia) trial [44]. This striking high prevalence among our studied cohort may be explained by nutritional factors specific to the Egyptian pediatric SCD population, especially those from lower socioeconomic backgrounds. The dietary pattern with limited access to nutrient-rich foods may significantly increase the risk of zinc deficiency in vulnerable groups such as children with SCD, who already have elevated zinc requirements. Besides, as a tertiary care center, we may be more likely to see clinically complex or more severely affected cases, potentially enriching our sample with children who have higher nutritional demands.

The baseline zinc levels in our studied cohort appeared to be inversely associated with the clinical indicators of disease severity; frequency of severe VOCs, frequency of hospital admissions due to severe VOCs, and infection rate; the higher the zinc level, the lower the frequency of these SCD complications [Fig. 1 (a, b, and c)]. Our findings were similar to several previous studies that documented the link between zinc deficiency in SCD patients and a variety of SCD-related complications (e.g., increased infection rate, VOCs, frequent hospital admissions, and faltered growth) [45–49]. Previous studies explained the contribution of zinc deficiency to the increased risk of VOCs in SCD patients because zinc deficiency increases gene expression and production of interleukin 1β (IL-1β) and tumor necrosis factor α (TNF-α). Additionally, zinc prevents TNF–α–induced endothelial cell damage, presumably by down-regulating oxidative stress-sensitive transcription factors [50, 51]. These cytotoxic cytokines are therefore generated in increased amounts from activated sickle cell monocytes, leading to the activation of endothelial cells, RBCs, and neutrophil adhesiveness, contributing to vascular inflammation, which is a key driver of vaso-occlusive phenomena in SCD [52].

In this study, the infection rate was higher at baseline and appeared to decrease after one year of zinc supplementation. Similarly, previous studies in adolescents and adults with SCD found that zinc supplementation reduced infection rates [27, 53]. These findings were explained previously by the role of zinc in supporting the immune system in many different ways. Zinc deficiency disrupts the normal development and function of innate as well as acquired immune cells, including macrophages, natural killer cells, and lymphocytes [54, 55]. Zinc acts as a second messenger of immune cells, and free zinc inside the cells is involved in signaling pathways and may be utilized as an antimicrobial agent by the innate immune cells [56–58].

In this study, the growth of the studied cases appeared to improve after one year of zinc supplementation. Furthermore, zinc supplementation was associated with a reduction in the incidence of VOCs and their severity, transfusion requirements, hospital admissions, and length of stays, which highlights the potential beneficial effects of zinc in amelioration of SCD phenotype and halting growth failure. Similarly, previous studies proved that zinc supplementation reduces the incidence of many SCD-related complications and improves the growth status [25–27, 53, 59, 60].

The anemia and hyperbilirubinemia also improved after oral zinc supplementation. Zinc improves the integrity of the RBCs, reducing the number of irreversibly sickled cells and increasing RBC survival, thus improving the anemia and ameliorating the hemolytic profile [61–63]. Zinc supplementation was also associated with improved oxidative stress markers in the studied SCD patients, likely by enhancing NO bioavailability and TAOA, reducing copper levels, and thereby potentially reducing oxidative stress. These findings have been supported by previous studies [20, 27]. Nitric oxide bioavailability is reduced in SCD and has been linked to the generation of ROS, contributing to oxidative damage. The ROS generated from auto-oxidation of HbS react with NO and form peroxynitrite, a highly reactive molecule, that further depletes the NO. Furthermore, ROS reduces the endothelial cells’ ability to produce NO [64, 65]. Serum ferritin also declined after oral zinc supplementation, possibly reflecting zinc’s anti-inflammatory effects, as well as its effect on reducing the transfusion requirements in SCD patients.

In this study, we observed a significant increase in the level of fetal hemoglobin in the studied SCD patients after zinc supplementation. This could be explained by the role of zinc in inducing the metal-responsive transcription factor-1 (MTF-1); one of the principal zinc metabolism regulators in higher eukaryotes through controlling the metal-inducible expression of metallothioneins and several other genes implicated directly in zinc’s intracellular sequestration and efflux transport. The MTF-1 also plays a role in hematopoiesis by activating gamma-globin gene expression [66]. Hydroxycarbamide remains the cornerstone of SCD management and the only approved fetal hemoglobin inducer [67]. Allowing only minimal hydroxycarbamide dose adjustments in our studied patients for weight changes or toxicities, we hypothesize that the beneficial effects of zinc in SCD could be further enhanced by gamma-globin gene expression and fetal hemoglobin induction. However, this hypothesis will need further future research.

## Conclusion

Zinc deficiency is highly prevalent in SCD children. Supplementation may offer clinical, hematological, and antioxidant benefits. Thus, zinc may be used as a routine supplement for zinc-deficient SCD patients.

### Strengths and limitations

This study has several notable strengths, addressing an important issue in SCD, which is zinc deficiency and its adverse clinical outcomes. Our study gives valuable insights into the potential therapeutic effects of oral zinc supplementation on the clinical, hematological, and oxidative status of SCD.

The absence of a control arm was a notable limitation of this study, as this was a single-arm study in which all participants received zinc supplementation, and there was no parallel group of untreated individuals. This design limits our ability to attribute observed changes solely to the intervention. Improvements in clinical and laboratory parameters could, in part, reflect natural growth, age- and/or time-related changes, or other unmeasured confounding factors. As a result, causal interpretations must be made with caution, and the observed benefits should be considered associative rather than definitively attributable to zinc supplementation. Despite this limitation, the study provides valuable real-world data on the prevalence of zinc deficiency and potential benefits of supplementation in this high-risk population. Future controlled or randomized trials are warranted to confirm these findings and further clarify the causal role of zinc in modifying clinical outcomes in SCD children.

Another limitation was the use of a small sample size, though convenient. More significant associations might have been yielded with a larger sample size. In addition, the study’s duration might have been insufficient to ascertain the long-term effects or possible adverse effects of zinc supplementation. We also recommend replicating this research with a larger sample of SCD patients in both steady and crisis states.

## Data Availability

No datasets were generated or analysed during the current study.
